# Misdiagnosis Based on Neoplastic Markers—Extremely High Alpha-Fetoprotein in Patients with Intrahepatic Cholangiocarcinoma with Literature Review of the Published Cases

**DOI:** 10.3390/medicina60071109

**Published:** 2024-07-09

**Authors:** Krzysztof Jakimów, Natalia Tekiela, Katarzyna Kozak, Robert Peterek, Anna Kwaśniewska, Jacek Pająk, Jerzy Chudek

**Affiliations:** 1 Student’s Scientific Association, Department of Internal Diseases and Oncological Chemotherapy, Medical University of Silesia, 40-055 Katowice, Poland; s76501@365.sum.edu.pl (N.T.); s81054@365.sum.edu.pl (K.K.); s76467@365.sum.edu.pl (R.P.); 2 Department of Radiology, The Mielecki Hospital, Medical University of Silesia, 40-055 Katowice, Poland; akwasniewska@onet.pl; 3 Department of Pathomorphology, Medical University of Silesia, 40-055 Katowice, Poland; jacek.pajak@sum.edu.pl; 4 Department of Internal Medicine and Oncological Chemotherapy, Medical University of Silesia, 40-055 Katowice, Poland

**Keywords:** alpha-fetoprotein, intrahepatic cholangiocarcinoma, histopathological examination

## Abstract

*Background*: Alpha-fetoprotein (AFP) and carbohydrate antigen 19-9 (CA 19-9) are two tumor markers that are widely used in the differential diagnosis in patients with primary liver tumors. Very high levels of AFP are sporadically observed in patients with intrahepatic cholangiocarcinoma (ICC) and may cause an incorrect initial diagnosis of hepatocellular carcinoma (HCC). *Methods*: Two cases of tumors in cirrhotic livers were described, in which the initial diagnosis, based on very high AFP levels (Patient I: 10,464 ng/mL, Patient II: 2212 ng/mL, reference range: ≤8.04 ng/mL) was HCC. In addition, the PubMed database was searched for cases of ICC with elevated AFP. *Discussion*: In both individuals, liver cirrhosis was diagnosed, but there was no typical rapid “washout” in the contrast-enhanced computed tomography. Based on the histological assessment of samples obtained in the core biopsies, the initially assumed diagnosis of HCC was changed to ICC in both cases. Only nine cases of patients with ICC and high AFP levels were found in the PubMed database. The AFP levels ranged from slightly elevated to over 16,000 ng/mL. *Conclusions*: A very high AFP level does not necessarily correlate with the presence of HCC. Therefore, the diagnosis has to be verified histologically, when the radiological imaging is uncertain in patients with liver cirrhosis.

## 1. Introduction

One of the most significant factors influencing the efficacy of primary liver cancer treatment is its early diagnosis. When radical surgical treatment cannot be applied, a correct diagnosis of the cancer type is essential before systemic therapy can be applied. The use of tumor markers, which may suggest the origin of the tumor, can facilitate the diagnostic process. Nevertheless, it is crucial to acknowledge that they do not exhibit the same high specificity observed in histological examinations and cannot replace pathological diagnoses.

Most tumor biomarkers approved by the Food and Drug Administration for clinical use are single serum-derived proteins [1]. Among them, alpha-fetoprotein (AFP) and carbohydrate antigen 19-9 (CA 19-9) are recognized as primary markers commonly utilized in the diagnostic procedure of liver tumors. AFP is a biomarker that has become widely used in the management of hepatocellular carcinoma (HCC). Its diagnostic role in primary liver tumors is limited as almost half of the patients with an early stage of the disease have serum AFP levels within the reference range [2,3]. It is important to notice that serum AFP concentration shows a poor correlation with the disease stage and up to 40% of patients may have it within the normal range [4]. Its values neither correlate with the tumor size nor the vascular invasion [5].

On the other hand, CA 19-9 is frequently utilized in the diagnosis of cholangiocarcinoma [6]. This marker is also physiologically present in the organism, and its elevation is not solely indicative of cholangiocarcinoma. It can also increase in other pathological conditions, such as pancreatic ductal adenocarcinoma, other adenocarcinomas of the digestive tract, or lung adenocarcinoma [7,8,9]. The serum CA 19-9 assessment alone has a sensitivity and specificity of 62% and 63% for diagnosing intrahepatic cholangiocarcinoma (ICC) [10]. Metastatic disease may be suspected if the concentration of CA 19-9 reaches above 1000 U/mL [11]. However, low values of CA 19-9 may also be misleading because 7–10% of the population do not secrete it and some malignancies may lose the ability to produce this marker [11,12,13,14,15].

HCC is considered the primary cause of death in patients with compensated liver cirrhosis. In this condition, screening for HCC may be associated with early detection and a chance of cure. Repeated abdominal ultrasonography in 6-month intervals is recommended as a cost-effective method that may be supplemented with the determination of AFP levels [16]. HCC is visualized as a hypo- or hyperechogenic lesion with increased blood flow and neovascularity. Ultrasound has a sensitivity reaching up to 87% and a specificity of up to 100%. In contrast-enhanced computed tomography (CT) examination HCC is typically characterized by an enhancement in the arterial phase and rapid washout in the portal venous phase. The sensitivity and specificity of CT are estimated at 65% and 96%, respectively [5].

Moderately elevated AFP values are also observed in 22.1–35.8% of patients with ICC alongside increased levels of CA 19-9 [17,18]. However, very high—over 50 times above the upper reference range—1000 ng/mL AFP levels are uncommon in the literature [10].

Both markers may also be associated with combined HCC–cholangiocarcinoma [19]. It is a rare entity with prevalence of 0.4–14.2% but in recent years, its incidence is rising. It shares symptomatology with HCC and cholangiocarcinoma. On imaging, it is similar to one of them depending on which component is predominant [20].

Herein, we present two patients who were diagnosed with tumors in cirrhotic livers with highly elevated AFP levels that raised the initial diagnosis of HCC. After extending the diagnostic work-up with radiological imaging and core biopsy, the diagnosis of ICC was established.

## 2. Materials and Methods

**Figure 1:**
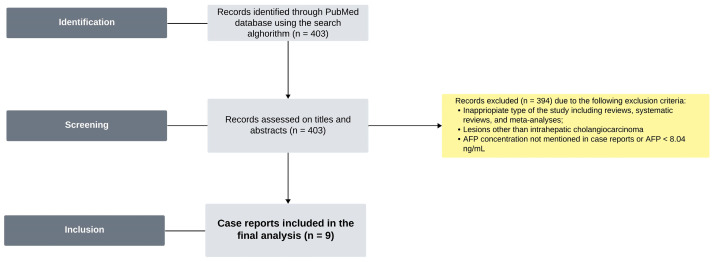
The flowchart for the study selection.

To find case report studies of ICC with elevated AFP, the PubMed database was searched using the following keywords: [(alpha-fetoprotein) OR (AFP)] AND [(ICC) OR (intrahepatic cholangiocarcinoma)]. In total 403 full-text articles were found. Papers that did not include AFP levels for individual patients were excluded. Finally, 9 studies were found to meet the following criteria: (1) ICC and (2) AFP > 8.04 ng/mL. The flow chart for study selection is presented in Figure 1.

## 3. Case Description

### 3.1. Patient I

**Figure 2:**
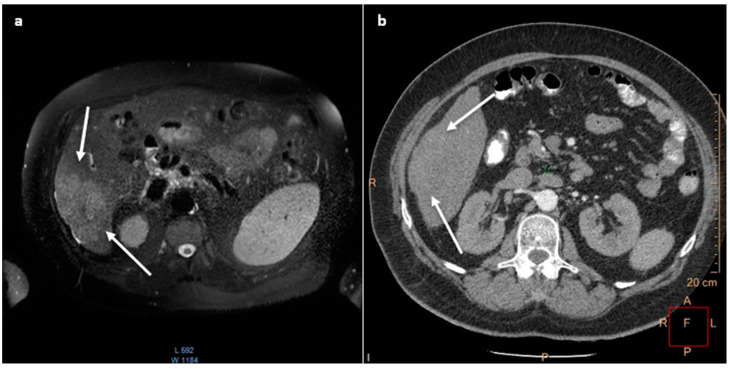
Axial magnetic resonance imaging (**a**) and computed tomography scan (**b**) of the abdomen visualizing a hypovascular tumor of the right lobe of the liver (white arrows) with no arterial enhancement.

A 61-year-old man was admitted to the Department for a core biopsy of a large right lobe liver tumor (90 × 70 × 77 mm) visualized in abdominal ultrasound, CT, and magnetic resonance imaging (MRI, Figure 2a), accompanied by liver cirrhosis and portal vein thrombosis. Despite the lack of typical radiological features of HCC in the CT, such as contrast washout (Figure 2b) [17], HCC was considered a potential diagnosis due to a significantly elevated AFP level (10,464 ng/mL; reference range: ≤8.04 ng/mL) and CA 19-9 (115 U/mL; reference range: ≤37 U/mL) to a lesser extent.

On admission, the patient complained of unintentional weight loss of 17 kg over the last 6-month period, decreased exercise tolerance, excessive weakness, and daytime sleepiness for about 2 months. The patient reported 3 colonoscopies with polypectomies performed within the last two years.

Toxic liver cirrhosis was scored as Child–Pugh class B reaching 7 points. Mild normocytic anemia and thrombocytopenia coexisted as indicators of the malfunction of the liver. In addition, laboratory tests revealed increased aspartate aminotransferase, gamma-glutamyl transpeptidase, lactate dehydrogenase activities, and elevated creatinine levels (in the course of co-existing chronic kidney disease).

**Figure 3:**
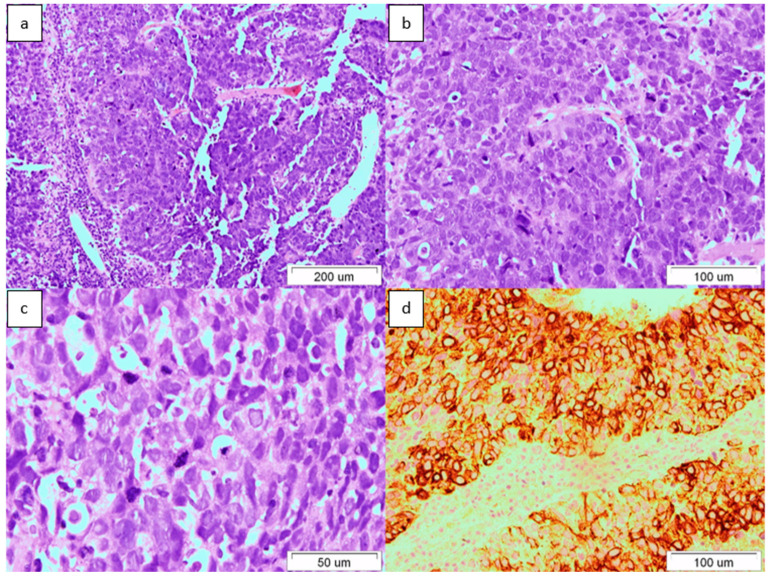
(**a**) G3 adenocarcinoma tissue; hematoxylin–eosin, original magnification 100×. (**b**) G3 adenocarcinoma tissue with numerous division figures; hematoxylin–eosin, original magnification 200×. (**c**) G3 adenocarcinoma tissue with numerous division figures; hematoxylin-eosin, original magnification 400×. (**d**) CD20 staining in cancer cells, original magnification 200×.

Ultrasound-guided core biopsy of the tumor was performed under local anesthesia. Unexpectedly pathological examination revealed low-differentiated foci of adenocarcinoma with immunohistochemical phenotype as follows: CK(+), CK20(+), CK7(−), PSA(−), CK19(−), CK18(+), AFP(−), hepatocytes(−), CDX2(−), CD56(+), synaptophysin(−), GTA3(−), CK5/6(−), p63(−), p40(−), CD57(−), TTF1(−), NSE(−). The microscopic image and immunohistochemical phenotype of the tumor were consistent with ICC (Figure 3).

### 3.2. Patient II

A 59-year-old woman with a history of obesity, type 2 diabetes, and liver cirrhosis in the course of non-alcoholic fatty liver disease was referred to the Department for a core biopsy of multiple nodular lesions in the liver. One month earlier, she was diagnosed with liver cirrhosis. The assessment was based on liver elastography which was scored as F4. Due to the tumor morphology in CT imaging and significantly elevated AFP levels in the range of between 1700 and 2212 ng/mL (reference range: ≤8.04 ng/mL), HCC was suspected. The level of carcinoembryonic antigen (CEA) was within the normal range (5.86 ng/mL; reference range ≤5 ng/mL).

On admission, the patient claimed a gradual deterioration in general condition, epigastric pain, and jaundice with pruritus. Physical examination revealed ascites. The patient was scored as Child–Pugh class B with 9 points.

A core biopsy of the nodular lesion was performed under local anesthesia with ultrasound guidance. Pathological examination revealed foci of low-differentiated adenocarcinoma suggesting the diagnosis of ICC rather than HCC.

## 4. Discussion

HCC and ICC are the two most common primary liver cancers [21,22]. AFP and CA 19-9 are markers that are widely in use in the differential diagnosis of HCC and ICC [11]. However, AFP, a typical biomarker, may be within the normal range in early non-cirrhotic or small HCC. Other markers that may be considered during the examination are des-gamma-carboxyprothrombin and lectin-bound AFP [5]. In the case of ICC, the features of cholestasis are frequently notable in laboratory tests, including the concentration of bound bilirubin, alkaline phosphatase, and γ-glutamyl transpeptidase activity. Nevertheless, the most significant is the increase in CA 19-9 levels [23]. However, 7–10% of the population do not secrete this marker. On the other hand, some malignancies may even lose the ability to produce it [11,12,13,14,15]. These conditions are likely to mislead the physicians.

Physiologically AFP is released into circulation by the fetal liver and yolk follicle [18,24], and its atypical elevation is frequently observed in patients suffering from HCC [2]. Soborczyk et al. [25] suggested that AFP levels > 500 ng/mL have almost 100% positive predictive value for HCC. It is noteworthy that increased levels of serum AFP are also associated with other clinical conditions such as hepatitis, liver failure or cirrhosis, ICC, gastric cancer, germ cell tumors, and inflammatory bowel ataxia telangiectasia [17,18]. A series of Japanese studies from a liver cancer center showed that 19% of patients with ICC had serum AFP levels > 20 ng/mL, 10.3% of them had >200 ng/mL, while serum AFP levels > 1000 ng/mL was recorded in 6.3% of the cases [26]. It is worth mentioning that differences in the sensitivity and specificity of this marker may occur depending on the chosen value of the cut-off point. Namely, at a cutoff value of 20 ng/mL, the sensitivity and specificity of AFP reach 41–65% and 80–94%, respectively [27]. At 20–100 ng/mL, sensitivity is 61% and specificity is 86%, while for 200 ng/mL, it is recorded as 49% and 98%, respectively [28]. In addition, high AFP values are prognostic for the worst overall survival and disease-free survival [29]. Interestingly, younger patients diagnosed with ICC and accompanying HBV infection tend to present higher AFP and lower CA 19-9 values [30]. This may implicate misdiagnosis in those patients as high AFP would raise a suspicion of HCC instead of ICC.

Radiological imaging plays a key role in the diagnostic process when HCC and ICC are suspected. Ultrasound, CT, and MRI are used during examination. The sensitivity of ultrasound reaches 51–87% and specificity ranges from 80% to 100% but is limited when a tumor is <2 cm. In this method, HCC is recognized as hypo- or hyperechogenic mass with increased blood flow and neovascularity. Contrast-enhanced ultrasound has both greater specificity and sensitivity, which reach up to 97% and 90%, respectively. In CT, HCC can be suspected when an enhancement in the arterial phase and rapid washout in the portal venous phase are seen. This imaging method has a sensitivity and specificity of 65% and 96%, respectively. However, for lesions < 2 cm, sensitivity decreases to 40%. The image obtained using MRI depends on how well-differentiated the tumor is. If the HCC is well-differentiated, it is hyperintense in T1 images and isointense in T2 sequence but when a poorly or moderately differentiated lesion is considered, it is isointense in T1 and hyperintense in T2. The sensitivity of MRI is 77–90%, while the specificity is between 84% and 97% [5]. As for the imaging studies used to make the diagnosis of ICC, conventional ultrasound, CT, MRI, and contrast-enhanced ultrasound are considered. MRI can be expected to provide a more accurate assessment of the primary tumor mass, while CT allows more precise detection of vascular enhancement. Initial arterial contrast enhancement at the tumor periphery, as well as progressive homogeneous contrast enhancement, can be expected on ICC imaging. The contrast-enhanced ultrasound method is more likely to misdiagnose ICC as HCC when compared with the methods described above. Therefore, it is not a reliable method in the differentiation of these tumors. Physicians should be aware of this in order to plan an optimal management of the pathology. On the other hand, it can be useful in situations with inconclusive CT or MRI. Of note, positron emission tomography imaging has limited accuracy in the day-to-day diagnosis of ICC, which is why it is usually not routinely used. Instead, 18F-fluorodeoxyglucose, can be useful for imaging metastatic lesions and lymph nodes being useful for the assessment of staging. Histological examination based on core biopsy is the standard for making a diagnosis of ICC. If the patient is suitable for resection, a biopsy is not necessary before radical surgery [31].

Patients presented in this report were referred for a core biopsy examination after disqualification from radical surgery. In the case of Patient I, it was the presence of a large lesion visualized in ultrasound, while in Patient II, it was cirrhosis with ascites followed by visualization of the lesion on imaging examination. Both patients had their tumor marker levels determined and both showed very high AFP titters of 10,464 ng/mL and 2212 ng/mL, respectively, raising the suspicion of HCC since values > 400 ng/mL are considered diagnostic for patients with liver cirrhosis [32]. As the radiological imaging was not specific for HCC, the histological examination of core biopsy was required to decide on palliative systemic therapy.

In the cirrhotic liver, ICC may rarely present with elevated levels of AFP. Moreover, increased CA 19-9 values may also occur in patients with HCC [33]. There is a limited number of ICC cases in the literature, that are presented with markedly elevated AFP levels. We have found only nine reports that met those criteria in the PubMed database. The records are presented in Table 1. The AFP levels in those patients ranged from slightly elevated to over 16,000 ng/mL. Therefore, clinicians should be aware of the possibility of enormously high AFP concentrations in non-HCC primary liver tumors. In a single cohort study conducted by Meng et al. [34], 7 out of 178 patients with ICC had AFP increased >200 ng/mL. The differences in the overall survival and disease-free survival were insignificant regarding the concentration of this marker. In another study by Hu et al. [35], among 70 ICC patients, only 2 (2.9%) had AFP >200 ng/mL.

Another point of interest, regarding the possible diagnosis, is tumors with a mixed structure of HCC and ICC. It is a rare malignancy with an incidence of 0.4–14.2% [20]. As a combination of two distinct tumors, it shares their characteristics regarding radiological imaging and laboratory tests. High AFP concentration has been recorded in 62.2% and CA 19-9 in 22.2%, while both markers were elevated together in 15% of cases. This condition may lead to the diagnosis of HCC due to the omission of histological examination in certain cases [19], raising the unanswered question of the number of core biopsies that should be taken from different parts of the tumor during the procedure.

An additional challenging factor is the occurrence of ICC in the cirrhotic liver. Cirrhosis was present in both described patients. This condition should be considered as a disabling factor of AFP as well as CA 19-9 in the differentiation of the mentioned tumors. It is usually found in the cases of HCC. On the other hand, ICC was once considered to appear in individuals without any signs of liver cirrhosis. Thus, this correlation was previously the differentiation method between HCC and ICC. In the cirrhotic liver, ICC can be visualized as a hypervascular lesion similar to HCC. Therefore, this finding may not distinguish those two tumors from each other. In MRI, the absence of contrast washout may not be a feature that helps differentiate ICC from HCC when a lesion develops in a cirrhotic liver. Hence, there is a possibility that a similar HCC enhancement pattern may appear at contrast-enhanced CT, MRI, or ultrasound [33]. All the above-mentioned information suggests that ICC detection in the cirrhotic liver during imaging makes diagnosis more challenging. Nevertheless, elevated AFP and CA 19-9 should raise a suspicion of a specific liver malignancy, but the proper diagnosis cannot be made depending solely on them. Thus, it is crucial to make the final diagnosis within a multidisciplinary team, especially when liver cancer is considered. The pathological examination of a specimen from the core biopsy is beneficial. This would help avoid misdiagnosing the patient without the typical features in imaging corresponding to neoplastic markers profile and prevent suboptimal or delayed treatment that is associated with decreased overall survival [43].

The small group of published cases included in this review should be mentioned as the main unavoidable study limitation.

## 5. Conclusions

Tumor markers are undoubtedly helpful for diagnosing and monitoring the progression and treatment response of primary liver cancers. However, they are not disease-specific, and even high concentrations of AFP may occur in patients with ICC. Histological examination is necessary to make the diagnosis in patients with liver cirrhosis but without the typical radiological image of HCC.

## Figures and Tables

**Table 1:** Characteristics of ICC patients with elevated alpha-fetoprotein levels.

Sex	Age	AFP Concentration [ng/mL]	Size of the Tumor in CT [mm]	Reference
Male	60	12,310	102 × 86 × 63	Wang et al. [30]
Male	48	10,602	88	Chun et al. [36]
Male	67	320	32	Chun et al. [36]
Female	67	15.7	94 × 98	Tasch et al. [37]
Female	63	28.7	70	Kashihara et al. [38]
Female	47	93	60	Nart et al. [39]
Male	55	3465	40	Park et al. [40]
Male	72	16,399	80 × 75 × 60	Yoh et al. [41]
Female	68	1561	NA	Zhang et al. [42]

AFP—alpha-fetoprotein, CT—computed tomography, NA—not available.

## Data Availability

Additional patient data can be obtained from the authors upon reasonable request.
